# The Need for Omics Studies in Chronic Kidney Disease of Unknown Etiology (CKDu): A Narrative Review and Perspective

**DOI:** 10.3390/ijms27135766

**Published:** 2026-06-26

**Authors:** Carly S. Chesterman, Amy S. Li, Chi-Yun Chen, Matthew Gibb, Richard J. Johnson, Zhoumeng Lin, Jared M. Brown

**Affiliations:** 1 Department of Pharmaceutical Sciences, Skaggs School of Pharmacy and Pharmaceutical Sciences, University of Colorado, Anschutz Medical Campus, Aurora, CO 80045, USA; carly.chesterman@cuanschutz.edu (C.S.C.);; 2 Division of Nephrology and Hypertension, Oregon Health and Science University, Portland, OR 97239, USA; amyli@ohsu.edu; 3 Department of Environmental and Global Health, College of Public Health and Health Professions, University of Florida, Gainesville, FL 32611, USA; 4 Center for Environmental and Human Toxicology, University of Florida, Gainesville, FL 32611, USA; 5 Division of Renal Diseases and Hypertension, University of Colorado, Anschutz Medical Campus, Aurora, CO 80045, USA

**Keywords:** CKDu, genomics, transcriptomics, metabolomics, proteomics, exposomics, artificial intelligence, machine learning

## Abstract

Chronic Kidney Disease of Unknown Etiology (CKDu) is an ongoing global health concern, particularly affecting agricultural communities in equatorial regions. Unlike traditional chronic kidney disease (CKD), CKDu occurs without common risk factors such as diabetes, hypertension, or kidney stones. Its etiology remains poorly understood, with environmental exposures, occupational hazards, and genetic susceptibility proposed as contributing factors. Omic technologies including genomics, transcriptomics, proteomics, metabolomics, and exposomics offer promising avenues to elucidate CKDu pathogenesis by enabling comprehensive molecular profiling and identification of biomarkers. Recent genomic studies have explored single nucleotide polymorphisms (SNPs) linked to kidney injury susceptibility, while transcriptomic analyses have identified differential expression of genes involved in oxidative stress and tubular injury pathways. Proteomic investigations have revealed candidate urinary biomarkers such as heat shock proteins and inflammatory mediators, and metabolomic profiling has highlighted alterations in amino acid and energy metabolism in affected individuals. Exposomic approaches are beginning to characterize cumulative chemical exposures, including pesticides and heavy metals, in endemic regions. This narrative review synthesizes current evidence on the application of omics approaches in CKDu research, highlights knowledge gaps, and proposes future directions for integrating multi-omics studies with machine learning and artificial intelligence approaches. Advancing omics-based investigations may provide critical insights into disease mechanisms, improve diagnostic precision, and inform targeted interventions for vulnerable populations.

## 1. Introduction

Chronic Kidney Disease of Unknown Etiology (CKDu) has emerged as an important and increasingly recognized global health concern primarily affecting equatorial agricultural communities. CKDu is defined as chronic kidney disease (CKD) occurring in the absence of traditional risk factors including diabetes, hypertension, nephrolithiasis, or other established causes of kidney disease [1,2,3]. Clinically, CKDu typically presents insidiously with a gradual decline in kidney function that disproportionately affects young to middle-aged agricultural workers especially in underdeveloped, under-resourced agricultural communities [2].

**Figure 1:**
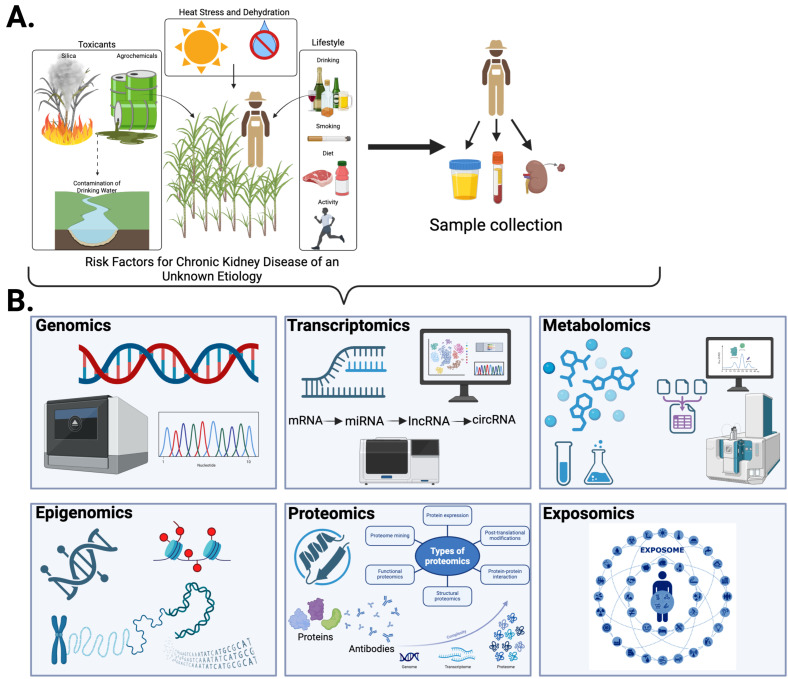
Potential risk factors for CKDu and various omics methods which can be utilized. (**A**) Summary of risk factors related to CKDu and biological samples worth collecting for further analysis. Multiple risk factors contribute to the pathogenesis of CKDu, including but not limited to recurrent heat stress and dehydration, environmental/occupational exposures (including agrochemicals, silica nanoparticles from sugar cane ash, or heavy metals). Invasive and non-invasive biologic samples including tissue from kidney biopsies, urine, and serum samples can be collected for further studies, including omics. (**B**) Summary of commonly utilized omics platforms, including genomics, epigenomics, transcriptomics, metabolomics, proteomics, and exposomics. Created with biorender.com.

Since its initial description in the 1990s among sugarcane workers in Central America and contemporaneous reports in northern Sri Lanka, other high-prevalence clusters have been described in other parts of Latin America, Sri Lanka, India, Thailand, and other subtropical regions [3]. Although initially enigmatic and elusive, observations in these CKDu hotspots have catalyzed major international research efforts, producing substantial progress in characterizing its epidemiology, clinical features, and mechanistic underpinnings over the past three decades. CKDu has been linked to environmental and occupational factors such as recurrent heat stress, dehydration, agrochemical exposures, and genetic susceptibility. Despite this, there remains significant heterogeneity in CKDu by region and pathology. CKDu has most commonly and classically been characterized by chronic tubulointerstitial nephritis and fibrosis, often with variable degrees of glomerulosclerosis that may represent a primary process or occur secondary to the kidney damage. For example, tubulointerstitial nephritis is the predominant biopsy finding in Sri Lanka [4]; however, there is rising evidence that focal segmental glomerulosclerosis is the predominant histologic finding in Aguascalientes, Mexico, a rising CKDu hotspot [5]. Ultimately, multiple risk factors likely interact with each other within vulnerable populations exposed to repeated physiologic and environmental stressors. The field has moved well beyond descriptive epidemiology and is increasingly focused on defining specific biomolecular pathways of injury. In this context, omics-based approaches, including genomics, transcriptomics, metabolomics, and other systems biology tools, offer a promising framework to unravel the heterogeneity of CKDu across regions, identify key risk factors for disease, and clarify causal mechanisms underlying disease risk and progression (Figure 1A).

Multi-omics approaches include the integration of a broad range of molecular datasets (genomics, transcriptomics, metabolomics, etc.) to provide a multidimensional view of biological processes (Figure 1B). Exposomics is an emerging field that expands on this framework by incorporating and studying cumulative environmental, dietary, and/or occupational exposures. The integration of multi-omics through advanced computational methods offers a powerful approach to refine clinical phenotype, identify early biomarkers of disease, and enhance our molecular understanding of the pathogenesis of CKDu.

Although the applications of omics to CKDu are still developing, important studies have already begun to generate clinically meaningful results and conclusions. Herein, we highlight the substantial progress made in understanding CKDu utilizing omics platforms, including disease profiling, mechanistic insights, and potential biomarkers of disease. Because this area of research may be unfamiliar to clinicians who frequently encounter CKDu, we briefly summarize the omics methodologies, discuss the important preliminary data to date, and discuss key assumptions and potential caveats in this narrative review. We also direct special attention to study design, population selection, exposure and outcome assessment, and computational integration by offering our perspectives and recommendations on how to utilize large omics datasets and machine learning approaches to improve our understanding of CKDu. Rather than reflecting limitations, these considerations may represent the natural progression of a maturing scientific discipline in this rapidly evolving field of CKDu and multi-omics. Our goal is to build upon the strong foundation of the current literature and propose a path forward utilizing machine learning-based integration of multi-omics datasets to further refine our understanding of CKDu, including disease classification, biomarker discovery, and therapeutic targeting.

## 2. Genomics

CKD is generally a complex, often polygenic disease involving many common genetic variants with estimated heritability varying between 30 and 75% [6,7]. Given the prevalence and complexity of CKDu in specific regions of the world, it is crucial to explore genetic risk factors that may predispose an individual to develop CKDu. Genomics originally was the study and determination of DNA sequences and has since evolved to include the functional analysis of how variation in DNA sequence affects phenotype at various levels of resolution. There are hypothesis-free methods, such as genome-wide association studies (GWAS), which scan the genome for common variants associated with disease. There are also hypothesis-driven methods, such as candidate gene analyses, that focus on a predefined biological pathway. Overall, genomics enables identification of inherited susceptibility and gene–environment interactions that predispose individuals to disease. In complex diseases such as CKDu, genomics can help distinguish individual and/or population-specific genetic risk factors and inform pathway-based hypothesis.

Genomic analyses represent the largest body of literature utilizing molecular technologies to elucidate the etiology and risk of developing CKDu, summarized in Table 1. Across four case–control studies spanning Sri Lanka, India, and Mexico, several genes implicated in CKDu include those related to renal tubular/ion transport (*SLC13A3*, a sodium-dependent transporter [8,9]; *KCNA10*, a potassium voltage-gated channel [9,10]), basement membrane integrity (*LAMB2*, a laminin protein [10]), and vascular regulation/signaling (*NOS3*, a endothelial nitric oxide synthase). While a more recent GWAS performed in a Nicaraguan population did not identify statistically significant variants for CKDu susceptibility, Native American ancestry was identified as a strong risk factor for CKDu [11]. They also identified a potentially protective variant in the *OPCML* gene associated with a substantially lower risk of CKDu due to potential role in fluid balance and thermoregulation [11]. Collectively, the genomics studies performed to date suggest that genetic variation may contribute to CKDu susceptibility. However, these findings remain preliminary and are limited by small sample sizes, lack of replication across diverse populations, and potential phenotype misclassification. As such, the role of inherited genetic risk in CKDu should be considered hypothesis-generating and requires further validation in larger, well-characterized cohorts.

Despite the advances made thus far in underscoring the genetic susceptibility of CKDu, several limitations constrain interpretation of these findings summarized in Table 1 and Table 2. The two major general limitations due to study design include relatively small cohorts, thereby limiting statistical power (especially for GWAS), and reliance on clinically diagnosed CKDu without kidney biopsy confirmation, increasing risk of phenotype misclassification. Importantly, technical aspects of each genomic platform also influence interpretation of the results (Table 2). Table 2 supplements the study summaries in Table 1 by providing a platform-level reference for interpreting the strengths and caveats of each genomic methodology and is intended to assist readers, particularly clinicians, who are less familiar with genomic methods, in evaluating the current literature and planning future studies. For example, GWAS is a powerful, unbiased, cost-effective platform that offers an excellent first-pass approach at identifying SNPs that confer increased (or decreased) risk of a disease. The basic assumption of GWAS is the common disease common variant hypothesis—that common diseases are caused by common variants (which may not always be the case), thus rare but clinically significant variants may be missed.

Importantly, unbiased platforms, such as GWAS, are heavily dependent on the quality of the reference genotyping array. In the first CKDu GWAS by Nanayakkara and colleagues in 2014 [8], they used the Illumina 500K chip, which was predominantly based on populations with European ancestry. While seminal at the time, genotyping arrays have since become far more genetically diverse (e.g., Illumina GDA), thus follow-up studies would be worthwhile. It is also worth noting that in the recent Nicaraguan CKDu GWAS by Friedman and colleagues in 2024 [11], the array used was the Illumina MEGA, which had <5% Native American background, thus may explain why a statistically significant variant was not identified in the cases. However, a major strength is that the authors inferred admixtures, which occur when genetic DNA is inherited from multiple ancestries, particularly Hispanic-American individuals who may have varying levels of Native American and/or Hispanic ancestry. This is ultimately a likely reason for why the study concluded that increasing Native American ancestry increases CKDu susceptibility.

Taken together, the genomic studies conducted to date converge on key inheritable traits that confer risk of CKDu including Native American ancestry, candidate disease variants genes involved in renal tubular ion transport *(SLC13A3* [8,9], *KCNA10* [9,10]), basement membrane integrity (*LAMB2* [10]), and nitric oxide signaling (*NOS3* [12]), and candidate protective variants involved in thermoregulation and fluid homeostasis (*OPCML* [11]). These findings correlate with known clinical features of CKDu, including geodemographic prevalence of disease, tubular dysfunction, and susceptibility to oxidative injury, particularly under heat stress and dehydration. However, the absence of biopsy-confirmed phenotypes and the use of genotyping arrays with limited non-European representation constrain the interpretability and generalizability of current findings. Additional studies are, therefore, needed for replication and validation, including fine-mapping and local ancestry analyses in various populations. Notably, no formal genomic meta-analysis or pooled analysis has yet been performed across the existing CKDu case–control cohorts due to insufficient sample sizes, variable genomic platforms and methods across studies, and varied definitions of cases and controls. A coordinated meta-analysis effort through the ISN i3C framework, as an example, with established methodologies [13] could substantially improve statistical power to detect and replicate common risk variants. Beyond validation and replication, additional gene–environment analyses, such as interaction models, Mendelian randomization, and family studies, are particularly important considering that environmental exposures have long been postulated to be important in the pathogenesis of CKDu. Finally, downstream mechanistic studies are needed to ascertain the role that candidate genes have on the pathogenesis and pathophysiology of CKDu. The field is better positioned to move from initial discovery toward integrated genomic analyses, deeper phenotyping, replications, and functional validation of candidate genes across diverse populations.

**Table 1:** Summary of genomic studies assessing genetic variants and risk of CKDu.

Author (Year) [Reference #], Country	Key Demographics	Methodology	Gene(s) of Interest	Conclusions/Implications	Major Limitations
Nanayakkara S, et al. (2014) [8], Sri Lanka	• *n* = 311 CKDu cases, 286 healthy controls • Men only, mean age 41–46 (yrs), agricultural workers • Established CKDu (mean SCr 2.1 mg/dL)	GWAS (Illumina 500 K array)	*SLC13A3* *OLFM3* *TMEM128* *LOC15727* *3*	1. A variant in the *SLC13A3* gene is associated with CKDu (odds ratio 2.13). 2. Heavy metals are not associated with CKDu.	• Illumina 500K array predominantly based on European population. • Possible selection bias (controls were not selected from a population with the same background as the cases) • No biopsy data.
Nanayakkara S, et al. (2015) [10], Sri Lanka	Same as above, but reduced to *n* = 301 cases, 276 controls (10 in each were excluded for quality control)	Whole exome	*PRCP* *LAMB2* *KNG1* *BANK1* *KCNA10* *SLC7A13* *TJP1*	1. *KCNA10* SNP is associated with developing CKDu (odds ratio 1.74). 2. Four rare variants in *LAMB2* were identified, of which three are novel variants found exclusively in cases.	• Possible selection bias (controls were not selected from a population with the same background as the cases) • No biopsy data.
Kumari R, et al. (2023) [9], India	• 3 groups, *n* = 60: CKD, CKDu, healthy control • % male: 60, 70, 57 • Mean age (yrs): 42, 47, 33 • Mean Scr (mg/dL): 8.3, 3.5, 0.8 • Occupation not reported.	Candidate gene/targeted SNP association study	*KCNA10* *SLC13A3*	1. *KCNA10* SNP is associated with CKDu susceptibility. 2. *SLC13A3* SNP is associated with both CKD and CKDu risk. 3. Specific genotypes are associated with higher CKDu risk.	• Small sample size. • *KCNA10* SNP in this study is different than the SNP identified in the above study. • No biopsy data.
Marín-Medina A, et al. (2023) [12], Mexico	• *n* = 105 CKDu cases, *n* = 90 heathy controls • % male: 80%, 71%, respectively • Mean age (yrs): 27, 24 • Mean SCr (mg/dL): 14.7, 0.5 • Occupation not reported.	Candidate gene/targeted SNP association study	*NOS3*	1. *NOS3* SNP (rs1799983) is protective against CKDu. 2. Another *NOS3* variant (rs2070744) is associated with higher odds of CKDu.	• Small sample size. • No biopsy data.
Friedman DJ, et al. (2024) [11], Nicaragua	• *n =* 429 CKDu cases, *n* = 385 healthy controls (discovery phase) • 98% male • Sugarcane farmers, miners, or brickmakers • SCr (mg/dL) for CKDu: ≥1.6 (male), ≥1.3 (female) • SCr (mg/dL) for control: ≤1.2 (male), ≤1.0 (female)	GWAS (Illumina MEGA)	*OPCML*	1. Native American ancestry is a strong risk factor for developing CKDu though no specific SNP associated with increased risk of CKDu in Native Americans was identified. 2. Healthy individuals with a rare variant in the *OPCML* gene exhibit higher urine osmolality and had 6-fold lower risk of developing CKDu. 3. *OPCML* knockout mice have altered fluid balance and temperature regulation, suggesting heat and dehydration are important risk factors for CKDu.	• Illumina MEGA array has <5% Native American background. • No biopsy data.

All studies were designed as case–control studies. Abbreviations: SCr, serum creatinine; GWAS, genome-wide association study; SNP, single nucleotide polymorphism.

**Table 2:** Summary of important genomic methods, their strengths, limitations, and key considerations in study design.

Platform/Method	Strengths	Limitations
Genome-wide association study (GWAS)	• Unbiased, hypothesis-free • Cost-effective • Best suited for detecting common variants	• Common disease common variant assumption that common diseases are influenced by common genetic variants • requires large sample sizes for statistical power • may miss rare but clinically significant variants • Reference databases and genotyping arrays predominantly based on European populations, increasing risk of misclassification of variants in non-European populations. • Identifies variants associated with disease but does not prove causality.
Whole exome sequencing (WES)	• Detects rare or novel variants in small cohorts or family studies • Higher resolution than GWAS (for variants in coding regions)	• Only detects variants in the protein-coding region (~1–2% of the genome). May miss clinically significant variants in noncoding regions. • More expensive than GWAS
Whole genome sequencing (WGS)	• Most comprehensive • Not limited by genotyping arrays • Gold-standard approach	• Most expensive method • High computational burden • Feasibility and scalability are limited due to cost and resources required
Candidate gene study	• Hypothesis-driven	• Hypothesis-driven methods may decrease detection of other genes/pathways not previously considered central to disease risk.
Key genomics study design components	Major considerations	
Sample size	Small sample sizes lead to underpowered analyses, false positive findings, and/or overestimated effect sizes (particularly in GWAS). Often, minimum recommended sample size is 100.	
Population of interest	Well-defined, pre-specific population limits generalizability to other populations but can also identify rare disease or protective variants unique to a specific population due to founder effects or genetic drifts, particularly pronounced in homogeneous, remote/isolated, or island populations (e.g., Sri Lanka).	
Admixed populations	Admixtures occur when genetic DNA is inherited from multiple ancestries, usually due to historical mixing events, such as colonization, migration, trade routes, slavery, or border changes (e.g., African-Americans, Hispanic-Americans). Admixtures can confound results of genomic analyses if not properly accounted for.	
Phenotype (clinical case definition)	Lack of kidney biopsy data and histopathologic information increases risk of phenotype misclassification.	

## 3. Epigenomics and Transcriptomics

Epigenetic modifications such as DNA methylation and histone modifications (e.g., methylation and acetylation) alter DNA and chromatin structures without directly changing the DNA sequence itself. These epigenetic modifications regulate patterns of gene expression, are often heritable, and can occur in response to exogenous or environmental exposures. Epigenomics evaluates these chemical modifications, which are often dynamic and reflect environmental or metabolic exposures, providing a bridge between external influence and sustained cellular adaptations. Whereas transcriptomics quantifies RNA expression, providing a snapshot of the transcribed genes at a given time point. Transcriptomics includes bulk and single-cell RNA sequencing approaches, identifying cell-type specific responses and dysregulated pathways. This method is particularly useful when there is a need to distinguish molecular phenotypes within clinically similar disease phenotypes; for example, in individuals at-risk for CKDu or early-stage CKDu.

The biological rationale for prioritizing epigenomic investigation in CKDu is well-supported by the broader kidney disease literature. Epigenetic dysregulation through aberrant DNA methylation and microRNA misexpression has emerged as a mechanistically important feature across a spectrum of renal conditions, from renal cell carcinoma, where promoter CpG island hypermethylation and microRNA dysregulation are well-established drivers of tumor suppressor silencing and disease progression [14], to non-malignant CKD, where methylation changes at genes regulating tubular fibrosis, oxidative stress, and inflammation have been identified as drivers of renal injury and progression [15]. This pattern of epigenetic sensitivity is particularly relevant to CKDu given that cadmium and arsenic, two nephrotoxic metals implicated in CKDu pathogenesis, are among the best-characterized environmental inducers of DNA methylation changes and altered microRNA expression in kidney proximal tubular cells, the primary cellular target of CKDu-associated injury [16,17].

To date, there is only one epigenomic study performed in CKDu [18] (Table 3), which suggested novel DNA methylation patterns in whole blood of Nicaraguan individuals with CKDu not previously reported in any other kidney disease, though none specifically correlated with disease onset. Interestingly, none of the methylation patterns were consistent with exposure to heat, pesticides, or select heavy metals, despite internal validation and comparison with known methylation patterns in smokers versus nonsmokers. Rightfully, the authors concluded that the DNA methylation profile should be re-examined in the future as epigenomic reference databases continue to improve.

Only three transcriptomic studies have been performed in CKDu. One study showed reduced expression of urinary microRNAs in a Sri Lankan cohort of CKDu patients and healthy endemic controls compared to healthy non-endemic controls, suggesting a common but unidentified exposure [19]. Two studies evaluated the transcriptome in whole blood samples from Sri Lankan individuals with CKDu [20,21], which demonstrated that inflammation and oxidative stress are increased in CKDu cases (Table 3). Importantly, these pathways are not unique to CKDu—inflammation, oxidative stress, and mitochondrial dysfunction are well-established features of CKD across multiple etiologies [22,23]. As such, current transcriptomic findings in CKDu likely reflect shared downstream injury responses rather than CKDu-specific mechanisms. Additionally, these studies utilized peripheral whole blood samples, which are prone to noise from multiple cell types and systemic signals that may be nonspecific to the kidney. While the initiating exposures in CKDu may differ, distinguishing exposure-specific molecular signatures from generalized kidney injury responses remains a key unresolved challenge.

Although the current epigenomic and transcriptomic studies are small and have not yet identified molecular signatures unique to CKDu, they provide important early insights into potential disease pathways, including inflammation, oxidative stress, and possible epigenetic/transcriptomic responses to environmental exposures in endemic areas. The finding that urinary microRNA downregulation occurs in both CKDu cases and endemic healthy controls, alongside novel DNA methylation patterns that do not correlate with known exposures, suggests that the epigenetic and transcriptomic landscapes in endemic populations may be shaped by environmental pressures that have yet to be fully characterized rather than disease itself. Critically, none of the current transcriptomic or epigenomic signatures have demonstrated discriminatory potential, meaning they cannot yet distinguish CKDu from other CKD etiologies or identify exposure-specific molecular fingerprints. Only one [20] of the four epigenomic/transcriptomic studies to date [18,19,20,21] included a CKD comparator; however none included longitudinal follow-up (all studies were cross-sectional) nor performed analyses such as receiver operating characteristic to assess diagnostic utility. Given that the predominant pathways identified, including inflammation, oxidative stress, and interferon signaling, are shared across CKD etiologies [22,23], the discriminatory value of current signatures is likely low in isolation. Future transcriptomic studies in CKDu should be explicitly designed with discrimination as a primary endpoint, incorporating well-characterized CKD comparator groups and statistical classifiers such as penalized logistic regression or random forests to identify exposure-specific or CKDu-specific transcriptomic features. Future studies must prioritize kidney tissue and cell-sorted urine samples over whole blood, incorporate well-matched CKD comparator groups, and be designed with sufficient statistical power to move beyond cataloging shared injury responses toward identifying what is molecularly distinct about CKDu.

**Table 3:** Summary of epigenomic and transcriptomic studies in CKDu.

Author (Year) [Reference #], Country	Key Demographics	Methodology	Specimen	Conclusions/Implications	Major Limitations
Oomatia A, et al. (2025) [18], Nicaragua	• 80+% male, mean age 25 yrs • *n* = 53 incident CKDu • *n* = 61 healthy controls • *n* = 16 established CKDu	Epigenome-wide association study of DNA methylation patterns (Illumina MethylationEPIC array)	Whole blood	• There are novel epigenetic changes in CKDu patients compared to healthy controls. • While these epigenetic modifications are novel, none were associated with disease onset. • They were also not associated with heat exposure, pesticides, arsenic, cadmium, chromium.	• Small sample size (but larger than others in its class). • No CKD control (unclear if epigenetic changes are CKDu-specific or nonspecific changes).
Sayanthooran S, et al. (2016) [20], Sri Lanka	• *n* = 43 CKDu • *n* = 14 CKD (DM or HTN) • *n* = 9 endemic healthy • *n* = 16 non-endemic healthy	Targeted gene expression profiling (*GCLC*, *GSTM1*, *G6PD*, *NLRP3*)	Whole blood	• *GCLC* is upregulated in CKDu, CKD, and endemic healthy compared to non-endemic healthy individuals; though *GCLC* is upregulated the least in CKDu. • *GSTM1* was not expressed in 25% of CKDu and nearly 50% of CKD patients. • *NLRP3* and *FGF23* are upregulated in both CKDu and CKD patients.	• Small sample size. • Panel of 5 oxidative stress genes assessed (limited assessment and technically not true ‘omics’ assessment). • Potential background noise from whole blood sample (results may not be kidney-specific). • Observational study, causality not established.
Sayanthooran S, et al. (2018) [21], Sri Lanka	• Discovery + validation cohort: all male, mean age 35–49 yrs • *n* = 24 + 30 CKDu (stage 2–5) • *n* = 6 + 10 endemic healthy • *n* = 6 + 10 non-endemic healthy	Whole transcriptomics (Illumina Human HT-12 v.4 array)	Whole blood	• Innate immunity (interferon and inflammasome signaling pathways) are upregulated in CKDu. • eIF2 and mTOR pathways are downregulated in CKDu. • Pathways linked to fluoride toxicity and infectious diseases are activated in CKDu patients, suggesting fluoride and infectious disease may be important contributors to developing CKDu.	• Small sample size. • No CKD control. • Potential background noise from whole blood sample. • Not stratified by severity/stage of CKDu. • Observational study; causality not established.
Edirithilake T, et al. (2023) [19], Sri Lanka	• All male, mean age 47 yrs • *n* = 3 endemic CKDu, mean eGFR 48 • *n* = 3 non-endemic healthy • *n* = 3 endemic healthy controls	MicroRNA transcriptomics (Illumina MiSeq)	Urine, first morning void	• Urinary small RNAs (microRNA and PIWI-interacting RNA) are downregulated in CKDu and endemic healthy controls compared to non-endemic healthy controls, suggesting the presence of an (still unidentified) environmental exposure driving epigenetic regulation. ^1^	• Very small sample size. • Limited demographic information; environmental exposures not systemically assessed or surveyed. • No CKD control, therefore currently without discriminatory potential. • Unclear if urinary small RNAs are biologically active and/or kidney-specific—6 of 10 identified small RNAs have no previously defined roles. • Unclear description of how RNA was isolated/purified. • Unclear description of normalization to urinary concentration or volume (if at all).

Abbreviations: DM, diabetes mellitus; HTN, hypertension; eIF2, Eukaryotic Initiation Factor 2; mTOR, mammalian target of rapamycin. ^1^ Four of the 11 microRNAs that were downregulated (namely, *miR-10b*, *miR-21*, *miR-148a*, and *miR-30a*) have also been shown to be linked with several environmental exposures including smoking [24], lead [25,26], arsenic [27,28], or bisphenol A (BPA) [29].

## 4. Metabolomics

The metabolome and proteome are highly dynamic, providing complementary insights into the downstream functional consequences of genetic and environmental influences by profiling small molecules and proteins, respectively. Metabolomics captures dynamic changes in metabolic pathways, reflecting real-time physiologic responses, while proteomics characterizes protein expression and modifications that mediate cellular function. Together, these are molecular technologies that can identify novel biomarkers and key biochemical pathways involved in various stages of disease, which can subsequently be used to guide diagnosis and/or treatment.

Three metabolomic studies have been performed on urine samples from Central American cohorts with normal kidney function (eGFR ≥ 75 mL/min/1.73 m^2^) but at risk of developing CKDu [1,30,31] (Table 4). Interestingly, despite differences in methodologies, these studies do share similar observations, namely perturbations in amino acid metabolism and central energy metabolism, including the kynurenine–tryptophan pathway, tricarboxylic acid (TCA) cycle intermediates, and nicotinamide metabolism. Similar metabolic disturbances have been observed in cross-sectional studies of the urine metabolome in CKD [32,33,34,35], raising questions about the specificity of the results to CKDu. Nevertheless, because these studies were conducted in individuals with preserved kidney function (eGFR ≥ 75 mL/min/1.73 m^2^), these findings may reflect exposure-related metabolic adaptations or early metabolic stress before the onset of clinical disease. While the urinary markers identified in these studies may have potential as indicators of exposure or early physiologic stress, their relevance to CKDu pathogenesis remains uncertain. However, because these studies are cross-sectional, it is unclear how many at-risk participants go on to develop CKDu. Those with the at-risk metabolic profile may not necessarily go on to develop CKDu; in fact, distinguishing why those at-risk did develop CKDu versus those who did not may offer rich insights into protective factors or risk factors. No study to date has assessed the metabolome in patients diagnosed with CKDu at any stage. Further investigation of the specificity and sensitivity of these biomarkers and validity of their clinical use is needed to determine its utility as a marker for risk of disease.

In fact, a major gap across all published CKDu metabolomic studies is the absence of formal discrimination analysis. No study has evaluated whether the urinary metabolomic profiles identified in at-risk or CKDu individuals can discriminate CKDu from other CKD etiologies or from healthy endemic controls using supervised classification approaches. This is a critical translational question: for a metabolomic biomarker to have clinical utility in CKDu, it must demonstrate adequate sensitivity and specificity in the relevant population. Future metabolomic studies should pre-specify discrimination as an outcome and apply appropriate analytical frameworks such as partial least squares-discriminant analysis, LASSO-based classifiers, or machine learning ensembles, with independent validation cohorts [36].

**Table 4:** Summary of metabolomic, proteomic, and exposomic studies in CKDu.

Author (Year) [Reference #], Country	Key Demographics	Methodology	Specimen	Conclusions/Implications	Major Limitations
Hall SM, et al. (2023) [30], Nicaragua	• *n* = 136, 43% male • Median age 19 yrs • At-risk adolescents and young adults, eGFR ≥ 90 (except for 8 where eGFR < 90 but not clinically diagnosed with CKD)	Metabolomics, untargeted (1H-NMR spectroscopy)	Urine, random (Baseline in 2011 and in 2015)	• There are also sex differences, primarily driven by metabolites in the citric acid cycle. • Urinary glycine concentrations are higher amongst individuals high-risk for CKDu • Urinary pyruvate is lower amongst individuals with lower eGFR • Individuals with lower eGFR exhibit declines in 1-methylnicotinamide and 2-oxoglutarate and increases in citrate and guanidinoacetate	• Cohort not followed longitudinally to determine which at-risk individuals developed incident CKDu. • The primary analysis remains cross-sectional, which limits causal inference and temporal understanding of metabolomic changes. • Key metabolite findings were not validated with targeted assays (like LC–MS) or functional biological assays.
Raines NH, et al. (2023) [31], Nicaragua	• *n* = 117 male sugarcane cutters • *n* = 78 male sugarcane workers (not cutters) • *n* = 78 Spanish male agricultural workers • *n* = 102 male miners or brickmakers At risk, GFR ≥ 75	Metabolomics, untargeted (1H-NMR spectroscopy)	Urine, end of shift (2015–2022)	• Urinary hippurate and other gut-derived metabolites are higher in male Nicaraguan sugarcane cutters/harvesters compared to other agricultural and nonagricultural workers • Urinary metabolites related to central energy metabolism are decreased in high risk individuals. • High-risk individuals exhibited higher kynurenate/tryptophan ratio, suggesting increased inflammation	• Unmatched, pairwise comparisons increase risk for false positive results. • Cohort not followed longitudinally to determine which at-risk individuals developed incident CKDu. • “At-risk” status was defined using biomarkers and clinical characteristics rather than confirmed CKDu diagnosis.
Stem AD, et al. (2024) [1], Guatemala	• total *n* = 20 male sugarcane cutters • *n* = 10 with baseline kidney function throughout the harvest season (mean eGFR 125) • *n* = 10 with at least 9% decline of kidney function throughout the harvest season (mean eGFR 123 à 103)	Metabolomics, untargeted (LCMS) Pesticide exposomics	Urine, morning pre-shift at the beginning and end of harvest season (paired urine samples)	• Urinary fatty acid and amino acid metabolites increase over the harvest season, which have been previously shown to be associated with impaired kidney function • Urinary silicon and certain pesticides increase over the harvest season in all sugarcane workers	• Small sample size. • Key occupational variables (e.g., heat stress, hydration status, workload intensity, infection, medication use) were not fully quantified or modeled. • Data were collected during a single harvest season, limiting insight into inter-annual variability or long-term exposure effects.
Kolli RT, et al. (2023) [2], Sri Lanka	• *n* = 23 male, non-endemic healthy, median age 50 yrs, not farmers • *n* = 24 male, endemic CKDu, median age 59 yrs, all farmers	Proteomics, untargeted (LC-MS/MS)	Urine, morning sample	• Urine albumin, cystatin C, and ß2-microglobulin are increased in CKDu, similar to other CKD in prior studies • Urine osteopontin, NGAL, and aquaporins are decreased in CKDu, contrary to other CKD in prior studies • The immune response, cell cycle (including apoptosis and cell death), and metabolic processes are most affected based on pathway analyses	• Modest number of patients and controls may reduce statistical power and sensitivity to detect smaller proteomic differences • Proteomic changes were not correlated with disease severity, progression, or clinical outcomes. • Detection limited by the sensitivity of mass spectrometry; low-abundance proteins and post-translational modifications may have been missed.

Abbreviation: neutrophil gelatinase-associated lipocalin (NGAL).

## 5. Proteomics

Proteomics is the comprehensive study of proteins and their roles in cellular functions, extending beyond protein identification to understand how protein pathways connect the extracellular environment to gene regulation [37]. Utilizing techniques such as mass spectrometry, proteomics can assess changes in protein expression, structure, and post-translational modifications [37,38]. Because proteins can be measured in easily accessible bodily fluids such as serum, urine, or saliva, proteomics hold strong potential for identifying early biomarkers of disease and elucidating important disease mechanisms in CKDu.

Kolli and colleagues were the first (and only) group to examine the urinary proteome profile of male Sri Lankan farmers with CKDu [2]. After comparison with CKD urinary proteome datasets, the CKDu urinary proteome was noted to be distinct, as demonstrated by reduced urinary osteopontin, neutrophil gelatinase-associated lipocalin (NGAL), and aquaporin levels [2]. They also noted that the CKDu urinary proteome exhibited similarities to patients with mitochondrial diseases, which is aligned with the in vitro metabolomics study that demonstrated mitochondrial dysfunction and altered energy pathways in HK-2 cells when exposed to silica nanoparticles derived from sugarcane ash [39]. Overall, these findings are consistent with a large body of prior literature that have associated renal dysfunction (both AKI and CKD) with mitochondrial dysfunction, including but not limited to abnormal morphology, increased oxidative stress, reduced ATP production, and reduced biogenesis [40,41,42]. Additional studies are needed to further characterize the mitochondrial profile and ascertain any unique features of mitochondrial dysfunction that may be specific to certain exposures associated with CKDu.

The proteomic findings described, particularly from Kolli and colleagues, highlight mitochondrial dysfunction and altered energy pathways as central features of CKDu, supported by a distinct urinary protein profile and suggesting unique disease mechanisms [2]. These observations are further reinforced by similarities to mitochondrial disease proteomic patterns and prior evidence linking renal injury with impaired mitochondrial function. Future proteomic studies should expand to larger human cohorts with well-characterized CKD comparators and apply validated classification frameworks to determine whether the CKDu urinary proteome carries clinically diagnostic information.

As with the metabolomic studies discussed above, a critical gap across the existing CKDu proteomic literature is the absence of formal discrimination analysis. The single human urinary proteomic study conducted to date [2] did not evaluate whether the identified proteomic profiles could distinguish CKDu from other CKD etiologies or from healthy individuals using classification approaches, and the pre-clinical studies are not designed to address this question directly. For urinary proteomic biomarkers to have clinical diagnostic utility in CKDu, future studies must be designed with discrimination as a primary endpoint, incorporating well-characterized CKD comparator groups and applying validated classification frameworks, with replication in independent cohorts to establish sensitivity and specificity in the relevant endemic populations.

## 6. Exposomics

Exposomics is the study of the cumulative environmental, dietary, and occupational exposures an individual encounters throughout their lifetime and how these exposures influence health and disease. By examining external factors such as pollutants, diet, stress, and lifestyle [43]—and integrating them with other omics technologies—exposomics enables identification of novel risk factors, exposure-related biomarkers, and a better understanding of gene–environment interactions. Exposures can be measured in a variety of ways, including wearable sensors, and coupled with high-resolution mass spectrometry as an example. Exposomics is particularly relevant to CKDu, where environmental exposures are strongly implicated. Integration with other omics platforms can ultimately support more precise and personalized prevention and treatment strategies [44].

Stem and colleagues (2024) exemplify this trend by combining elemental analysis and untargeted metabolomics to characterize the occupational exposome of Guatemalan sugarcane workers across a harvest season [1]. They identified key exposures including silica, pesticide metabolites (e.g., paraquat and carbofuran), but not heavy metal uptake, are associated with disrupted energy metabolism, fatty acid accumulation, and amino acid perturbations, all consistent with early metabolic stress in the kidneys. By connecting external exposures to internal biochemical changes, this work underscores the power of exposome-metabolome integration for biomarker discovery and mechanistic insight. This approach aligns with current exposomic priorities to move beyond single exposure paradigms toward holistic, systems level assessments of exposure disease relationships.

An additional strength of exposomic studies is a meaningful shift from many other CKDu studies that often rely on self-reported exposures (such as smoking) which has been demonstrated to underestimate true exposure prevalence [45]. In CKDu-endemic agricultural communities, accurate recall of exposure dose, duration, specific chemical agents, and seasonal variability is inherently limited by factors including low literacy rates, language barriers in migrant worker populations, and the practical difficulty of distinguishing residential from occupational exposures in communities [46,47]. Thus, use of self-reported data increases risk of misclassification and recall bias, thereby limiting the accuracy of exposure outcome associations. The study by Stem and colleagues (2024) [1] exemplifies this principle directly within a CKDu context, using paired urinary metabolomics and exposomic analysis to objectively characterize silica and pesticide exposures across a full harvest season without reliance on workers’ exposure recall. Future studies should build on this model by combining biospecimen collection with community-level environmental sampling and passive personal exposure monitors such as wearable silicone samplers, to triangulate exposure estimates across multiple independent measurement approaches. These multi-source strategies, when integrated with internal omics data, will substantially improve the reliability and biological specificity of exposure-disease associations in CKDu research.

Despite these advances, several important limitations remain. Firstly, this study by Stem et al. is based on a single-harvest-season study. Agrochemical use in these regions is highly variable across harvest seasons and years. Individuals are typically exposed to complex mixtures of environmental and occupational factors, making it difficult to isolate the effects of individual exposures versus mixtures. Secondly, the cross-sectional designs limit causal inference, and reverse causation remains a concern, as impaired kidney function may itself alter metabolomic or biochemical profiles. As such, while exposomic approaches provide valuable insights into exposure–biology relationships, longitudinal and intervention-based studies are needed to establish causal links between specific exposures and CKDu development.

An underappreciated dimension of exposomics research in CKDu is the broader public health context in which it must operate. CKDu predominantly affects agricultural laborers in low- and middle-income countries, including Nicaragua, El Salvador, Guatemala, Sri Lanka, and India, where population-level public health surveillance infrastructure is often limited or absent [48,49]. Occupational exposure registries, pesticide use databases, and environmental monitoring networks are frequently incomplete or inaccessible for research purposes. This creates a fundamental asymmetry: the populations most affected by CKDu have the least public health data available. Exposomic research in these settings must, therefore, be designed not only to generate scientific insights but also to build local capacity for longitudinal public health surveillance. Community-based participatory approaches, integration with existing agricultural health monitoring programs, and co-generation of data with local ministries of health and occupational health agencies represent promising strategies to address this gap [50]. Ultimately, the findings of exposomic studies will only translate into meaningful public health action if local health systems have the infrastructure to act on them.

The emerging exposomic literature in CKDu represents a meaningful conceptual advance in how the field approaches exposure characterization. By moving beyond questionnaire-based exposure assessment toward objective biospecimen-based quantification of the chemical burden carried by agricultural workers, it has been demonstrated that the exposome–metabolome interface is both measurable and biologically informative in this population [1]. The silica, pesticide, and metabolic signatures identified are coherent with the broader pattern of disrupted energy metabolism and tubular stress that emerges across the metabolomic and proteomic literature, suggesting that occupational chemical exposures are plausibly upstream drivers of the molecular perturbations observed in at-risk individuals. Realizing the full potential of exposomics in CKDu will require multi-season designs, broader chemical panels, and integration with the public health infrastructure of endemic regions to move from characterizing exposure to establishing causation.

## 7. Integrating Machine Learning and Artificial Intelligence with Multiple Types of Data

Machine learning (ML) and artificial intelligence (AI) approaches have been extensively adopted in clinical practice for medical diagnosis and disease prediction, including nephrology and kidney disease assessment [51,52,53]. Emerging studies have applied ML techniques to CKDu prevalence prediction and early-stage detection on local and national scales, which show promise [54,55]. While these approaches provide valuable insights into population-level patterns and potential risk factors, they are largely limited to external or exposome-related features. This constraint restricts the ability of current models to capture underlying biological mechanisms, which are critical for distinguishing CKDu from other forms of CKD and for elucidating disease initiation and progression.

Multi-omics approaches are reshaping disease classification, prognosis, and mechanistic understanding across a wide range of complex diseases [56,57]. By characterizing biological processes across multiple molecular layers, multi-omics data offer an unprecedented opportunity to elucidate disease pathways, identify biomarkers, and distinguish disease subtypes that may not be apparent from clinical or environmental data alone. In CKDu, where the etiology remains unclear and likely multifactorial, multi-omics profiling may provide critical insights into underlying biological perturbations and gene–environment interactions.

**Figure 2:**
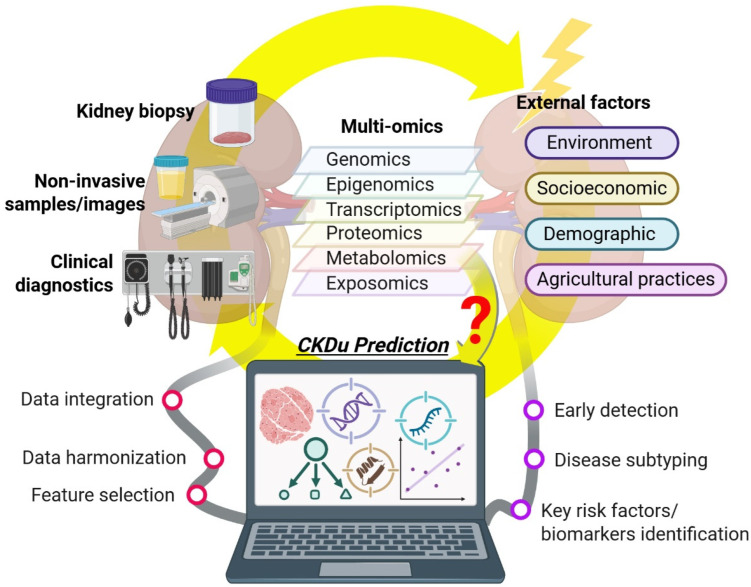
Schematic illustration of machine learning–based multi-omics integration incorporating heterogeneous data types to support CKDu research. Created with biorender.com.

Several studies have employed single-omics or multi-omics datasets to identify biomarkers associated with CKD [58,59,60,61,62]. However, the integration of ML with multi-omics data remains a promising yet underexplored direction in CKDu research. By jointly analyzing complementary molecular layers, ML-based integrative approaches can enhance model interpretability, improve predictive performance, and elucidate complex molecular pathways that are often inaccessible through single-omics analyses. Figure 2 illustrates an ML-driven framework for integrating multi-omics data with heterogeneous data sources to support CKDu prediction and characterization. Individual-level information can be derived from invasive and non-invasive biological samples, including kidney biopsy, blood and urine specimens, imaging data, and routine clinical diagnostics, generating multiple omics layers [63,64]. These internal biological data are complemented by external factors, which collectively contribute to CKDu risk. Advanced AI methods, such as feature selection algorithms, network-based models, and deep learning architectures, are well-suited to handle the dimensionality, sparsity, and complexity inherent in multi-omics data [65].

To integrate multi-level omics datasets, both vertical and horizontal integration frameworks have been proposed [66]. Additionally, neural network-based deep learning approaches, especially hierarchical stacked autoencoders, have shown strong performance in learning latent representations within individual omics layers and capturing complex cross-omics interactions [67,68]. When further combined with epidemiological and environmental information, these integrative frameworks have the potential to enable more accurate disease subtyping, facilitate earlier detection, and support the identification of candidate biomarkers and mechanistic pathways underlying CKDu (Figure 2). It is important to emphasize that the ML-based multi-omics integration framework described here and illustrated in Figure 2 represents a prospective, future-oriented conceptualization.

However, applying machine learning to CKDu presents several unique challenges. Available datasets to date are small relative to the high dimensionality of multi-omics data, increasing the risk of overfitting and limiting generalizability. Cohort heterogeneity across geographic regions, exposure profiles, and clinical definitions of CKDu further complicates model development and validation. Beyond the challenge of limited sample sizes, robust multi-omics integration in CKDu research will require careful attention to several analytical pre-processing considerations that become complex in a cross-omics context. Within each omics layer, normalization should be tailored to the data type: urine metabolomics often uses creatinine correction or probabilistic quotient normalization to handle dilution, with added variance stabilization for NMR data [69]; transcriptomic data require library size normalization such as TMM or DESeq2 variance-stabilizing transformation [70]; and genomic data require principal component adjustment for population stratification. When integrating these layers, an additional challenge arises because data from different omics platforms are measured on inherently distinct scales and exhibit varying distributional characteristics, necessitating careful harmonization prior to joint modeling. Batch effects present a further and often underappreciated complication, arising from differences in sample processing, laboratory site, platform version, or collection date, and are becoming especially problematic in CKDu research. Established tools such as ComBat address batch effects within individual omics layers [71], but cross-omics batch harmonization remains an active area of methodological development [72]. Finally, missing data are ubiquitous in multi-omics studies, arising from analyte-specific detection limits and inconsistent coverage across platforms. For metabolomics specifically, random forest imputation has been shown to perform best for missing completely at random and missing at random data types, while quantile regression imputation of left-censored data is preferred for values below detection limits [73]. Across all pre-processing steps, explicit and transparent reporting of methods is essential for reproducibility and will be critical as CKDu multi-omics datasets are harmonized across sites and research groups.

## 8. Discussion, Challenges, and Final Recommendations for Future Omics Studies in CKDu

An underexplored opportunity within the existing CKDu literature is secondary integrative analysis aimed at identifying shared molecular signatures across published datasets. Despite differences in geography, methodology, and sample type, the studies reviewed here show notable convergence: disrupted central energy metabolism and amino acid pathways appear across multiple independent metabolomic cohorts from Nicaragua and Guatemala [1,30,31]; inflammation and oxidative stress emerge consistently across transcriptomic datasets from Sri Lanka [20,21]; and mitochondrial dysfunction is implicated by both urinary proteomics in Sri Lanka [2]. These overlapping signals represent candidate shared biomarkers that have not yet been formally evaluated across datasets in an integrated framework. Cross-dataset harmonization and re-analysis using integrative tools such as MOFA+ (multi-omics factor analysis) or DIABLO (data integration analysis for biomarker discovery using latent components) could extract shared latent factors of disease biology and begin to distinguish core CKDu molecular features from region-specific or exposure-specific signals [74,75]. Such analyses are partially constrained by the current absence of publicly deposited raw data, and we therefore encourage future authors to deposit omics datasets in accessible repositories such as Gene Expression Omnibus, MetaboLights, and PRIDE to enable this work.

As noted above, advances in understanding CKDu have come a long way, but the single overarching question remains: what are the main drivers of disease? A critical first step toward answering this question is accurate phenotyping through the incorporation of kidney biopsies. The ISN i3C Working Group recommends routine kidney biopsy in any individual suspected to have CKDu and that the decision to biopsy should be a shared decision between patient and provider that includes a cost–benefit discussion such as current landscape of limited treatments, potential costs and complications to the patient [76,77]. Only through histopathologic characterization can CKDu be precisely defined as a heterogeneous disease with variable glomerular and/or tubulointerstitial pathology, which could reflect different etiologies. If CKDu represents a spectrum of diseases rather than a single entity, research approaches need to be tailored accordingly.

Incorporation of kidney biopsy data into CKDu studies represents one of the most important opportunities to advance the field. Where feasible, kidney biopsies in well-characterized research cohorts—particularly among individuals at-risk or with early-to-moderate-stage CKDu—may provide critical insight into disease heterogeneity and underlying pathology. However, practical, ethical, and resource constraints in many endemic regions necessitate careful consideration of risk–benefit tradeoffs and may limit widespread implementation. For research purposes, longitudinal biopsy strategies may provide critical insights into disease incidence, progression, and role of environmental exposures. For example, one could consider obtaining a biopsy at baseline (or pre-harvest) in individuals with or without clinical CKDu, post-exposure (or end of the harvest season), and at long-term follow up (e.g., in 1–5 years). As outlined in The International Society of Nephrology’s (ISN) i3C CKDu Toolkit, incorporation of kidney biopsy within well-characterized research cohorts, particularly in early disease, represents an important opportunity to strengthen CKDu phenotyping and support integration of molecular and exposure based analyses, while accounting for ethical, practical, and resource constraints [78].

In addition to histologic phenotyping, rigorous study design with well-matched populations is essential across all omics approaches. Study cohorts should be balanced by age, sex, occupation, residential location (endemic vs non-endemic), and ideally include individuals with CKDu, healthy controls (from endemic and non-endemic regions), and individuals with other CKD. Given the longstanding hypothesis that environmental exposure holds a central role, exposure-based stratification is key, including at-risk individuals (exposed but without clinical CKD), exposed individuals with CKDu), and unexposed controls from endemic and non-endemic regions. Longitudinal follow-up of at-risk individuals is particularly important to determine which individuals ultimately develop CKDu as early molecular profiles may not directly translate to clinical disease; this observation itself could also shed light on key protective or risk factors in the pathogenesis of CKDu.

Clinical staging is another key component of rigorous study design and analysis. Several CKDu studies included in this review have pooled multiple stages together without subgroup analysis likely due to small sample sizes. However, early-stage CKD (stage 3A and above) is generally expected to have a different biochemical profile than late-stage CKD (stage 4 or 5), where fibrosis, progressive uremia, and systemic inflammation are dominant features and may obscure other or early disease-specific signals. Thus, focusing on individuals at-risk or with early-to-moderate stage CKDu (stages 1–3B) in omics studies are likely of highest value. Nevertheless, studying CKDu in advanced stages (4 and 5) can play an important role to discover any potential distinct biomarkers are present into the late stages for diagnosis and treatment monitoring purposes and could shed light on shared disease pathways with other etiologies of CKD, such as fibrogenic pathways.

Across omics platforms, sample selection, timing, and processing are critical determinants of data quality and interpretability. Genomic data are largely invariant across time and tissues, whereas epigenomic, transcriptomic, metabolomic, proteomic, and exposomic profiles are highly context-specific and tissue- or cell-specific. Kidney tissue, when available, provides the most direct insight into disease biology, and should be prioritized, including single-cell and/or spatial analyses. Urine is often felt to contain more kidney-specific data; however the supernatant is most often studied, and the urinary pellet is frequently discarded. Thus, we also propose that both urine supernatant and pellet be preserved as the pellet may represent an underutilized source of cellular and molecular data for omics platforms. Complementary sampling of blood and urine—ideally collected simultaneously with tissue—can provide a comprehensive snapshot in time. For non-genomic studies (i.e., epigenomics, transcriptomics, metabolomics, proteomics, and exposomics), longitudinal and exposure-informed sampling strategies are particularly important. Serial sampling of blood and urine at key time points—such as at the beginning and end of work shifts or harvest seasons—can capture dynamic biochemical responses to environmental exposures or stressors that could reveal early, treatment-responsive biomarkers that precede detectable kidney function decline with serum creatinine or cystatin C. However, careful interpretation is required to ascertain if these biomarkers are evidence of a truly physiologic adaptation or if the response is maladaptive, especially with recurrent insults. Long-term follow-up, including after removal from putative exposures, is also essential to define the natural history of CKDu and determine reversibility of the injury/disease.

Replication, validation, and generalizability remain fundamental challenges across all omics studies. Genomic findings require additional follow-up studies including fine-mapping, local ancestry analysis, and functional gene validation. Epigenomic, transcriptomic, metabolomic, and proteomic studies should assess if identified profiles are exposure-specific, stage-dependent, and/or distinct from other forms of CKD, recognizing that the interpretability of all omics findings is inherently dependent on the quality of the reference databases used (such as HMDB and KEGG for metabolomics, UniProt for proteomics, and ENCODE and Roadmap Epigenomics for epigenomics), which continue to improve but still contain gaps in functional annotations, particularly for non-European populations. Currently published omics signatures, particularly transcriptomics and epigenomics [18,19,20,21], in CKDu have not yet been demonstrated to be unique to a particular exposure. However, building these profiles is key foundational work that can be compared to signatures associated with known exposures. Finally, amassing these omics profiles with putative exposures can be utilized and integrated to develop a risk assessment tool [79].

Because of the complex and dynamic nature of omics datasets, integration of machine learning with these datasets will be increasingly important to capture a holistic view of what is occurring in various stages of CKDu from at-risk to clinical disease. Despite the great potential of applying machine learning approaches to advance CKDu research, several methodological challenges remain. These include limited sample sizes, missing omics data, and methodological constraints of harmonizing data across different omics platforms. In addition, substantial imbalances in feature dimensionality across omics layers may bias model training, as algorithms tend to preferentially learn from datasets with larger numbers of variables, potentially overlooking informative signals from lower-dimensional omics modalities [67]. Finally, further advances in feature selection strategies and model interpretability are needed to fully realize the potential of machine learning-driven multi-omics integration in CKDu research.

There has been substantial progress in understanding CKDu as a disease entity since its initial description in Sri Lanka during the 2000s, yet much remains to be learned and discovered. The ISN i3C CKDu Toolkit provides a unifying framework to improve the quality, equity, and comparability of CKDu research by emphasizing standardized case definitions, harmonized data and biospecimen collection, ethical use of kidney biopsy, and robust community engagement in high burden settings. Within this framework, integrated application of genomics, epigenomics, transcriptomics, metabolomics, proteomics, and exposomics is essential to refine disease phenotypes, elucidate gene environment interactions, and identify molecular pathways underlying CKDu risk and progression. Taken together, these recommendations establish a practical framework to guide CKDu research toward more precise disease classification, deeper mechanistic insight, and effective prevention approaches.

## 9. Conclusions

Omics-based approaches are uniquely positioned to advance our mechanistic understanding of CKDu, but the field remains in an early, hypothesis-generating phase. The studies reviewed here collectively support that CKDu involves convergent molecular perturbations in energy metabolism, oxidative stress, ion transport, and inflammation. Genomic studies implicate heritable susceptibility in ion transport and fluid homeostasis pathways; transcriptomic and epigenomic studies suggest that inflammation and oxidative stress are early molecular responses in endemic populations; metabolomic and proteomic analyses point toward disrupted mitochondrial function and perturbed amino acid metabolism as early markers of renal stress; and exposomic studies are beginning to objectify the chemical burden borne by at-risk workers. However, many findings remain preliminary, limited by small sample sizes and cross-sectional designs, lacking replication across diverse populations, and have not yet demonstrated CKDu-specific discriminatory value. The field’s next critical step is to move from descriptive cataloging to integrative, longitudinal, adequately powered, and biopsy-anchored multi-omics studies, ideally conducted within the harmonized framework of the ISN i3C CKDu Toolkit, with the explicit goal of distinguishing CKDu-specific molecular signatures from generalized CKD injury responses, and ultimately translating omics discoveries into early detection tools and targeted interventions for vulnerable populations.

## Data Availability

No new data were created or analyzed in this study. Data sharing is not applicable to this article.

## References

[1] Stem A.D., Brindley S., Rogers K.L., Salih A., Roncal-Jimenez C.A., Johnson R.J., Newman L.S., Butler-Dawson J., Krisher L., Brown J.M. (2024). Exposome and Metabolome Analysis of Sugarcane Workers Reveals Predictors of Kidney Injury. Kidney Int. Rep..

[2] Kolli R.T., Gunasekara S.C., Foster M.W., Adduri S., Strasma A., Wyatt C., Konduru N.V., De Silva M.C.S., Jayasundara N. (2023). The urinary proteome infers dysregulation of mitochondrial, lysosomal, and protein reabsorption processes in chronic kidney disease of unknown etiology (CKDu). Am. J. Physiol. Ren. Physiol..

[3] Johnson R.J., Wesseling C., Newman L.S. (2019). Chronic Kidney Disease of Unknown Cause in Agricultural Communities. N. Engl. J. Med..

[4] Pett J., Linhart C., Osborne N., Morrell S., Fahim M., Knight J., Premaranthne S., Wazil A.W.M., Ratnatunga N., Wijethunga S. (2024). Tubulointerstitial nephropathy is the predominant finding in men in a review of more than 3000 renal biopsies over a 10-year period from Sri Lanka. BMC Nephrol..

[5] Gutierrez-Pena M., Zuniga-Macias L., Marin-Garcia R., Ovalle-Robles I., Garcia-Diaz A.L., Macias-Guzman M.J., Delgado-Bentites A., Macias-Diaz D.M., Prado-Aguilar C.A., Vega de la Rosa A. (2021). High prevalence of end-stage renal disease of unknown origin in Aguascalientes Mexico: Role of the registry of chronic kidney disease and renal biopsy in its approach and future directions. Clin. Kidney J..

[6] Yu Z., Jin J., Tin A., Kottgen A., Yu B., Chen J., Surapaneni A., Zhou L., Ballantyne C.M., Hoogeveen R.C. (2021). Polygenic Risk Scores for Kidney Function and Their Associations with Circulating Proteome, and Incident Kidney Diseases. J. Am. Soc. Nephrol..

[7] Tin A., Kottgen A. (2020). Genome-Wide Association Studies of CKD and Related Traits. Clin. J. Am. Soc. Nephrol..

[8] Nanayakkara S., Senevirathna S.T., Abeysekera T., Chandrajith R., Ratnatunga N., Gunarathne E.D., Yan J., Hitomi T., Muso E., Komiya T. (2014). An integrative study of the genetic, social and environmental determinants of chronic kidney disease characterized by tubulointerstitial damages in the North Central Region of Sri Lanka. J. Occup. Health.

[9] Kumari R., Tiwari S., Atlani M., Anirudhan A., Goel S.K., Kumar A. (2023). Association of Single Nucleotide Polymorphisms in KCNA10 and SLC13A3 Genes with the Susceptibility to Chronic Kidney Disease of Unknown Etiology in Central Indian Patients. Biochem. Genet..

[10] Nanayakkara S., Senevirathna S.T., Parahitiyawa N.B., Abeysekera T., Chandrajith R., Ratnatunga N., Hitomi T., Kobayashi H., Harada K.H., Koizumi A. (2015). Whole-exome sequencing reveals genetic variants associated with chronic kidney disease characterized by tubulointerstitial damages in North Central Region, Sri Lanka. Environ. Health Prev. Med..

[11] Friedman D.J., Leone D.A., Amador J.J., Kupferman J., Francey L.J., Lopez-Pilarte D., Lau J., Delgado I., Yih W.K., Salinas A. (2024). Genetic risk factors for Mesoamerican nephropathy. Proc. Natl. Acad. Sci. USA.

[12] Marin-Medina A., Gomez-Ramos J.J., Mendoza-Morales N., Figuera-Villanueva L.E. (2023). Association between the Polymorphisms rs2070744, 4b/a and rs1799983 of the NOS3 Gene with Chronic Kidney Disease of Uncertain or Non-Traditional Etiology in Mexican Patients. Medicina.

[13] Winkler T.W., Day F.R., Croteau-Chonka D.C., Wood A.R., Locke A.E., Mägi R., Ferreira T., Fall T., Graff M., Justice A.E. (2014). Quality control and conduct of genome-wide association meta-analyses. Nat. Protoc..

[14] Morris M.R., Latif F., Morris M.R., Latif F. (2016). The epigenetic landscape of renal cancer. Nat. Rev. Nephrol..

[15] Kato M., Natarajan R., Kato M., Natarajan R. (2019). Epigenetics and epigenomics in diabetic kidney disease and metabolic memory. Nat. Rev. Nephrol..

[16] Guo A.H., Kumar S., Lombard D.B. (2022). Epigenetic mechanisms of cadmium-induced nephrotoxicity. Curr. Opin. Toxicol..

[17] Reichard J.F., Puga A. (2010). Effects of arsenic exposure on DNA methylation and epigenetic gene regulation. Epigenomics.

[18] Oomatia A., Chervova O., Al-Rashed A.M., Smpokou E.T., Ecker S., Pearce N., Heggeseth B., Nitsch D., Cardenas A., Beck S. (2025). Longitudinal leucocyte DNA methylation changes in Mesoamerican nephropathy. Environ. Epigenetics.

[19] Edirithilake T., Nanayakkara N., Lin X.X., Biggs P.J., Chandrajith R., Lokugalappatti S., Wickramasinghe S. (2023). Urinary MicroRNA Analysis Indicates an Epigenetic Regulation of Chronic Kidney Disease of Unknown Etiology in Sri Lanka. Microrna.

[20] Sayanthooran S., Magana-Arachchi D.N., Gunerathne L., Abeysekera T.D., Sooriyapathirana S.S. (2016). Upregulation of Oxidative Stress Related Genes in a Chronic Kidney Disease Attributed to Specific Geographical Locations of Sri Lanka. Biomed. Res. Int..

[21] Sayanthooran S., Gunerathne L., Abeysekera T.D.J., Magana-Arachchi D.N. (2018). Transcriptome analysis supports viral infection and fluoride toxicity as contributors to chronic kidney disease of unknown etiology (CKDu) in Sri Lanka. Int. Urol. Nephrol..

[22] Kishi S., Nagasu H., Kidokoro K., Kashihara N. (2024). Oxidative stress and the role of redox signalling in chronic kidney disease. Nat. Rev. Nephrol..

[23] Speer T., Dimmeler S., Schunk S.J., Fliser D., Ridker P.M. (2022). Targeting innate immunity-driven inflammation in CKD and cardiovascular disease. Nat. Rev. Nephrol..

[24] Vrijens K., Bollati V., Nawrot T.S. (2015). MicroRNAs as Potential Signatures of Environmental Exposure or Effect: A Systematic Review. Environ. Health Perspect..

[25] de Araújo M.L., Gomes B.C., Devóz P.P., Duarte N.d.A.A., Ribeiro D.L., Araújo A.L.d., Batista B.L., Antunes L.M.G., Barbosa F., Rodrigues A.S. (2021). Association Between miR-148a and DNA Methylation Profile in Individuals Exposed to Lead (Pb). Front. Genet..

[26] Kong A.P.S., Xiao K., Choi K.C., Wang G., Chan M.H.M., Ho C.S., Chan I., Wong C.K., Chan J.C.N., Szeto C.C. (2012). Associations between microRNA (miR-21, 126, 155 and 221), albuminuria and heavy metals in Hong Kong Chinese adolescents. Clin. Chim. Acta.

[27] Polo A., Marchese S., De Petro G., Montella M., Ciliberto G., Budillon A., Costantini S., Polo A., Marchese S., De Petro G. (2018). Identifying a panel of genes/proteins/miRNAs modulated by arsenicals in bladder, prostate, kidney cancers. Sci. Rep..

[28] Concessao P.L., Prakash J. (2025). Arsenic-induced nephrotoxicity: Mechanisms, biomarkers, and preventive strategies for global health. Vet. World.

[29] Kim J.H., Cho Y.H., Hong Y.-C. (2020). MicroRNA expression in response to bisphenol A is associated with high blood pressure. Environ. Int..

[30] Hall S.M., Raines N.H., Ramirez-Rubio O., Amador J.J., Lopez-Pilarte D., O’Callaghan-Gordo C., Gil-Redondo R., Embade N., Millet O., Peng X. (2023). Urinary Metabolomic Profile of Youth at Risk of Chronic Kidney Disease in Nicaragua. Kidney360.

[31] Raines N.H., Leone D.A., O’Callaghan-Gordo C., Ramirez-Rubio O., Amador J.J., Lopez Pilarte D., Delgado I.S., Leibler J.H., Embade N., Gil-Redondo R. (2023). Metabolic Features of Increased Gut Permeability, Inflammation, and Altered Energy Metabolism Distinguish Agricultural Workers at Risk for Mesoamerican Nephropathy. Metabolites.

[32] Kim Y., Lee J., Kang M.S., Song J., Kim S.G., Cho S., Huh H., Lee S., Park S., Jo H.A. (2023). Urinary Metabolite Profile Predicting the Progression of CKD. Kidney360.

[33] Steinbrenner I., Schultheiss U.T., Kotsis F., Schlosser P., Stockmann H., Mohney R.P., Schmid M., Oefner P.J., Eckardt K.U., Kottgen A. (2021). Urine Metabolite Levels, Adverse Kidney Outcomes, and Mortality in CKD Patients: A Metabolome-wide Association Study. Am. J. Kidney Dis..

[34] Liu J.J., Liu S., Zheng H., Lee J., Gurung R.L., Chan C., Lee L.S., Ang K., Ching J., Kovalik J.P. (2025). Urine Tricarboxylic Acid Cycle Metabolites and Risk of End-stage Kidney Disease in Patients with Type 2 Diabetes. J. Clin. Endocrinol. Metab..

[35] Lopez-Hernandez Y., Oropeza-Valdez J.J., Maeda-Gutierrez V., Zheng J., Mandal R., Lopez-Ramos J.E., Moreira Hernandez J.C., Jaime-Sanchez E., Romo-Garcia M.F., Enciso Moreno J.A. (2025). Comprehensive and quantitative urinary metabolomic profiling for improved characterization of diabetic nephropathy. Metabolomics.

[36] Gromski P.S., Muhamadali H., Ellis D.I., Xu Y., Correa E., Turner M.L., Goodacre R. (2015). A tutorial review: Metabolomics and partial least squares-discriminant analysis—A marriage of convenience or a shotgun wedding. Anal. Chim. Acta.

[37] Dominguez D.C., Lopes R., Torres M.L. (2007). Introduction to proteomics. Clin. Lab. Sci..

[38] Petricoin E.F., Liotta L.A. (2003). Clinical applications of proteomics. J. Nutr..

[39] Stem A.D., Rogers K.L., Roede J.R., Roncal-Jimenez C.A., Johnson R.J., Brown J.M. (2023). Sugarcane ash and sugarcane ash-derived silica nanoparticles alter cellular metabolism in human proximal tubular kidney cells. Environ. Pollut..

[40] Che R., Yuan Y., Huang S., Zhang A. (2014). Mitochondrial dysfunction in the pathophysiology of renal diseases. Am. J. Physiol. Ren. Physiol..

[41] Galvan D.L., Green N.H., Danesh F.R. (2017). The hallmarks of mitochondrial dysfunction in chronic kidney disease. Kidney Int..

[42] Jiang M., Bai M., Lei J., Xie Y., Xu S., Jia Z., Zhang A. (2020). Mitochondrial dysfunction and the AKI-to-CKD transition. Am. J. Physiol. Ren. Physiol..

[43] Wan M., Simonin E.M., Johnson M.M., Zhang X., Lin X., Gao P., Patel C.J., Yousuf A., Snyder M.P., Hong X. (2025). Exposomics: A review of methodologies, applications, and future directions in molecular medicine. EMBO Mol. Med..

[44] Vineis P., Robinson O., Chadeau-Hyam M., Dehghan A., Mudway I., Dagnino S. (2020). What is new in the exposome?. Environ. Int..

[45] Butler-Dawson J., Barnoya J., Brindley S., Krisher L., Fan W., Asensio C., Newman L.S. (2021). Cross-sectional study examining the accuracy of self-reported smoking status as compared to urinary cotinine levels among workers at risk for chronic kidney disease of unknown origin in Guatemala. BMJ Open.

[46] Rappaport S.M., Smith M.T. (2010). Environment and Disease Risks. Science.

[47] Wild C.P. (2005). Complementing the Genome with an “Exposome”: The Outstanding Challenge of Environmental Exposure Measurement in Molecular Epidemiology. Cancer Epidemiol. Biomark. Prev..

[48] Wesseling C., Crowe J., Hogstedt C., Jakobsson K., Lucas R., Wegman D.H. (2014). Resolving the Enigma of the Mesoamerican Nephropathy: A Research Workshop Summary. Am. J. Kidney Dis..

[49] Almaguer M., Herrera R., Orantes C.M. (2014). Chronic kidney disease of unknown etiology in agricultural communities. MEDICC Rev..

[50] Correa-Rotter R., Wesseling C., Johnson R.J. (2014). CKD of Unknown Origin in Central America: The Case for a Mesoamerican Nephropathy. Am. J. Kidney Dis..

[51] Battineni G., Sagaro G.G., Chinatalapudi N., Amenta F. (2020). Applications of Machine Learning Predictive Models in the Chronic Disease Diagnosis. J. Pers. Med..

[52] Sabanayagam C., Banu R., Lim C., Tham Y.C., Cheng C.Y., Tan G., Ekinci E., Sheng B., McKay G., Shaw J.E. (2025). Artificial intelligence in chronic kidney disease management: A scoping review. Theranostics.

[53] Sanmarchi F., Fanconi C., Golinelli D., Gori D., Hernandez-Boussard T., Capodici A. (2023). Predict, diagnose, and treat chronic kidney disease with machine learning: A systematic literature review. J. Nephrol..

[54] Lokuarachchi D.N., Manoj J.T., Weerasooriya M.N.H., Waseem M.N.M., Aslam F., Kumarasinghe N., Kasthurirathne D. (2020). Prediction of CKDu using KDQOL score, Ankle Swelling and Risk Factor Analysis using Neural Networks. 2020 2nd International Conference on Advancements in Computing (ICAC).

[55] Rajapaksha N., Rajawasan H., Ubeysinghe R., Perera S., Ravi Supunya Swarnakantha N.H.P., Gamage M., Nanayakkara N., Wijayakulasooriya J., Herath D., Lakmali M. (2025). An integrated data-driven approach for chronic kidney disease of unknown etiology (CKDu) risk profiling and prediction in Sri Lanka. Seventh International Conference on Information Technology and Computer Communications (ITCC 2025), Yokohama, Japan, 2025.

[56] Chen C., Wang J., Pan D., Wang X., Xu Y., Yan J., Wang L., Yang X., Yang M., Liu G.P. (2023). Applications of multi-omics analysis in human diseases. MedComm.

[57] Mani S., Lalani S.R., Pammi M. (2025). Genomics and multiomics in the age of precision medicine. Pediatr. Res..

[58] Hirakawa Y., Yoshioka K., Kojima K., Yamashita Y., Shibahara T., Wada T., Nangaku M., Inagi R. (2022). Potential progression biomarkers of diabetic kidney disease determined using comprehensive machine learning analysis of non-targeted metabolomics. Sci. Rep..

[59] Sha Q., Lyu J., Zhao M., Li H., Guo M., Sun Q. (2020). Multi-Omics Analysis of Diabetic Nephropathy Reveals Potential New Mechanisms and Drug Targets. Front. Genet..

[60] Tuechler N., Burtscher M.L., Garrido-Rodriguez M., Khan M.M., Turei D., Tischer C., Kaspar S., Schwarz J.J., Stein F., Rettel M. (2025). Dynamic multi-omics and mechanistic modeling approach uncovers novel mechanisms of kidney fibrosis progression. Mol. Syst. Biol..

[61] Wu I.W., Tsai T.H., Lo C.J., Chou Y.J., Yeh C.H., Chan Y.H., Chen J.H., Hsu P.W., Pan H.C., Hsu H.J. (2022). Discovering a trans-omics biomarker signature that predisposes high risk diabetic patients to diabetic kidney disease. npj Digit. Med..

[62] Zhou X.J., Zhong X.H., Duan L.X. (2023). Integration of artificial intelligence and multi-omics in kidney diseases. Fundam. Res..

[63] Eddy S., Mariani L.H., Kretzler M. (2020). Integrated multi-omics approaches to improve classification of chronic kidney disease. Nat. Rev. Nephrol..

[64] Zhang M., Ye Z., Yuan E., Lv X., Zhang Y., Tan Y., Xia C., Tang J., Huang J., Li Z. (2024). Imaging-based deep learning in kidney diseases: Recent progress and future prospects. Insights Imaging.

[65] Yuan S., Guo L., Xu F. (2025). Artificial intelligence in nephrology: Predicting CKD progression and personalizing treatment. Int. Urol. Nephrol..

[66] Wu C., Zhou F., Ren J., Li X., Jiang Y., Ma S. (2019). A Selective Review of Multi-Level Omics Data Integration Using Variable Selection. High Throughput.

[67] Picard M., Scott-Boyer M.P., Bodein A., Perin O., Droit A. (2021). Integration strategies of multi-omics data for machine learning analysis. Comput. Struct. Biotechnol. J..

[68] Xu J., Wu P., Chen Y., Meng Q., Dawood H., Dawood H. (2019). A hierarchical integration deep flexible neural forest framework for cancer subtype classification by integrating multi-omics data. BMC Bioinform..

[69] Parsons H.M., Ludwig C., Günther U.L., Viant M.R., Parsons H.M., Ludwig C., Günther U.L., Viant M.R. (2007). Improved classification accuracy in 1- and 2-dimensional NMR metabolomics data using the variance stabilising generalised logarithm transformation. BMC Bioinform..

[70] Love M.I., Huber W., Anders S., Love M.I., Huber W., Anders S. (2014). Moderated estimation of fold change and dispersion for RNA-seq data with DESeq2. Genome Biol..

[71] Johnson W.E., Li C., Rabinovic A. (2007). Adjusting batch effects in microarray expression data using empirical Bayes methods. Biostatistics.

[72] Leek J.T., Scharpf R.B., Bravo H.C., Simcha D., Langmead B., Johnson W.E., Geman D., Baggerly K., Irizarry R.A., Leek J.T. (2010). Tackling the widespread and critical impact of batch effects in high-throughput data. Nat. Rev. Genet..

[73] Wei R., Wang J., Su M., Jia E., Chen S., Chen T., Ni Y., Wei R., Wang J., Su M. (2018). Missing Value Imputation Approach for Mass Spectrometry-based Metabolomics Data. Sci. Rep..

[74] Argelaguet R., Arnol D., Bredikhin D., Deloro Y., Velten B., Marioni J.C., Stegle O., Argelaguet R., Arnol D., Bredikhin D. (2020). MOFA+: A statistical framework for comprehensive integration of multi-modal single-cell data. Genome Biol..

[75] Singh A., Shannon C.P., Gautier B., Rohart F., Vacher M., Tebbutt S.J., Lê Cao K.-A. (2019). DIABLO: An integrative approach for identifying key molecular drivers from multi-omics assays. Bioinformatics.

[76] Wijewickrama E., Behera S., Garcia P., Avila-Casado C., Caplin B., Paolo V.S., Courville K., Friedman D., Madero M., Jha V. (2024). Kidney biopsies among persons living in hotspots of CKDu: A position statement from the International Society of Nephrology’s Consortium of Collaborators on CKDu. Kidney Int..

[77] Caplin B., Anand S., Gonzalez-Quiroz M., Madero M., Michael M., Mohan S., Strasma A., Swaminathan S., Waikar S., Wijewickrama E. (2026). Taking the “unknown” out of CKDu-optimizing approaches to uncover the cause(s) of epidemic-level kidney disease in low- and middle-income settings: A report from the ISN’s International Consortium of CKDu Collaborators (ISN i3C). Kidney Int..

[78] Catalina Alvarez-Elias A., Wijewikrama E., Caplin B. ISN i3C (CKDu) Toolkit. https://www.theisn.org/initiatives/toolkits/isn-i3c-ckdu-toolkit/#contact.

[79] Cecchetto M., Peruzza L., Giubilato E., Bernardini I., Rovere G.D., Marcomini A., Regoli F., Bargelloni L., Patarnello T., Semenzin E. (2023). An innovative index to incorporate transcriptomic data into weight of evidence approaches for environmental risk assessment. Environ. Res..

